# Combined Channel Attention and Spatial Attention Module Network for Chinese Herbal Slices Automated Recognition

**DOI:** 10.3389/fnins.2022.920820

**Published:** 2022-06-13

**Authors:** Jianqing Wang, Weitao Mo, Yan Wu, Xiaomei Xu, Yi Li, Jianming Ye, Xiaobo Lai

**Affiliations:** ^1^School of Medical Technology and Information Engineering, Zhejiang Chinese Medical University, Hangzhou, China; ^2^First Affiliated Hospital, Gannan Medical University, Ganzhou, China

**Keywords:** automated recognition, intelligent data analysis, artificial intelligence, spatial attention module, computational intelligence

## Abstract

Chinese Herbal Slices (CHS) are critical components of Traditional Chinese Medicine (TCM); the accurate recognition of CHS is crucial for applying to medicine, production, and education. However, existing methods to recognize the CHS are mainly performed by experienced professionals, which may not meet vast CHS market demand due to time-consuming and the limited number of professionals. Although some automated CHS recognition approaches have been proposed, the performance still needs further improvement because they are primarily based on the traditional machine learning with hand-crafted features, resulting in relatively low accuracy. Additionally, few CHS datasets are available for research aimed at practical application. To comprehensively address these problems, we propose a combined channel attention and spatial attention module network (CCSM-Net) for efficiently recognizing CHS with 2-D images. The CCSM-Net integrates channel and spatial attentions, focusing on the most important information as well as the position of the information of CHS image. Especially, pairs of max-pooling and average pooling operations are used in the CA and SA module to aggregate the channel information of the feature map. Then, a dataset of 14,196 images with 182 categories of commonly used CHS is constructed. We evaluated our framework on the constructed dataset. Experimental results show that the proposed CCSM-Net indicates promising performance and outperforms other typical deep learning algorithms, achieving a recognition rate of 99.27%, a precision of 99.33%, a recall of 99.27%, and an F1-score of 99.26% with different numbers of CHS categories.

## Introduction

Chinese herbal medicine is the foundation of Traditional Chinese Medicine (TCM), where Chinese Herbal Slices (CHS), or herbal pieces, are the key components (Hua and Chung, 2015). CHS are made after special concocted processes from Chinese herbal medicine, and then are widely employed in the treatment of diseases, with a result that the quality and accurate usage of CHS become extremely important. Although it plays such an important role in the TCM, in practical CHS production, sale, and dispensing, their recognition and identification are still being processed almost entirely by human based on their professional experience. Moreover, there are hundreds of categories of commonly used CHS, and as they come from herbs and have undergone a series of processing, that their specificity in texture, color, and shape have been weakened, which makes them tend to be confused with each other. Therefore, the accuracy of identification depends very much on the people’s subjective judgment and experience level. As a result, the accuracy and stability of identification are difficult to be guaranteed.

**FIGURE 1:**
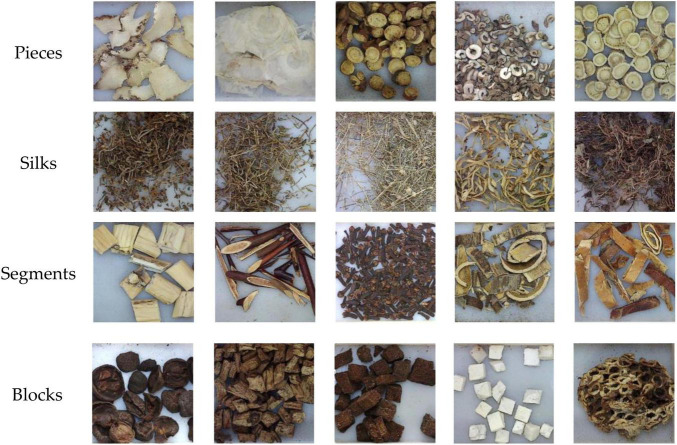
Different shape types of Chinese herbal slices (CHS).

**FIGURE 2:**
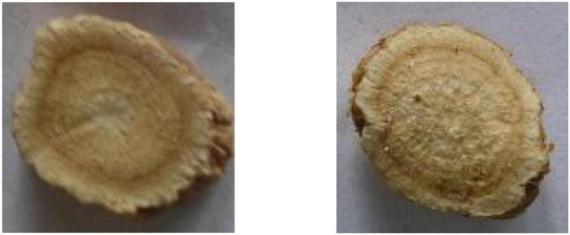
Examples of easily confused CHS. Astragali radix **(left)** and sophorae flavescentis radix **(right)**.

With the fast development of computer technology and the application of artificial intelligence, the automated recognition for CHS has emerged. It can be used to monitor and track the production process of CHS, transform the traditional experience description of CHS identification into quantitative evaluation indicators, and assist to improve the automation and intelligence level of TCM production equipment. In computer-aided CHS classification, machine learning and deep learning-based image recognition is most effective and reliable. However, CHS has its unique characteristics, making its recognition different from other recognition problems in image recognition. Generally speaking, only in term of the shape, CHS can be divided into many different types, such as pieces, silks, segments, and blocks, as shown in Figure 1. There are great differences in shape between different types. Then it is more appropriate to adopt global features (Kabbai et al., 2019) in inter-category classification. However, many CHS from the same type are very similar in shape, color, and texture, which make them difficult to be accurately distinguished even for experienced professionals, such as astragali radix and sophorae flavescentis radix, as shown in Figure 2. Then, local features are comparatively better for the identification. Therefore, it is unlikely to use unified features or features with approximate scales to identify all categories of CHS. In addition, due to the differences lie in the CHS processing standards, methods, and producing areas, there are few large professional public datasets available for the research and application of CHS recognition.

In this paper, a combined channel attention and spatial attention module network (CCSM-Net) integrating both channel attention (CA) and spatial attention (SA) is proposed to realize better recognition of CHS images. Furthermore, a local CHS image dataset is constructed, including commonly used CHS of various shapes as well as color and texture types as previously described. The main contributions of this paper are as follows:

(1) A CCSM-Net is proposed to focus on both the most important information as well as the position of the information on CHS images, to capture more detailed position and channel information for better CHS classification.
(2) A pair of max-pooling and average-pooling operation is used in the SA module to aggregate the channel information of the feature map. The modules can efficiently integrate features with accurate channel and spatial information.
(3) A new CHS image dataset including commonly used CHS images of different categories is proposed to realize image recognition for this research, and match the actual scenario of CHS production and dispensing.

The rest of this paper is organized as follows. Section “Deep learning for the Chinese herbal slices image recognition” describes the relevant deep learning research on CHS recognition and elaborates the proposed method in detail, including the CCSM and the pooling design. Then the CHSD dataset and experimental results are presented and discussed in Section “Analytical approaches and results.” Final conclusions are summarized in Section “Conclusion.”

## Deep Learning for the Chinese Herbal Slices Image Recognition

### Deep Learning

Deep neural networks are conducive for classification and feature visualization (Alam et al., 2020; Jing and Tian, 2021). The recognition results on large datasets such as ImageNet (Russakovsky et al., 2015), MS-COCO (Lin et al., 2014), or CIFAR (Krizhevsky, 2009) are generally better than that of traditional algorithms based on low-level hand-crafted features. In deep learning models, the convolutional neural network (CNN) extracts features hierarchically from a global perspective for image description (Hu et al., 2018), and it has been widely studied and applied in image recognition (Schmidhuber, 2015; Gu et al., 2018). Many deep learning models, such as AlexNet (Krizhevsky et al., 2012), VGGNet (Simonyan and Zisserman, 2014), GoogLeNet (Szegedy et al., 2015), ResNet (He et al., 2016), Res2Net (Gao et al., 2019; Wu et al., 2022), and FPN (Liu et al., 2021; Xu et al., 2021; Zeng et al., 2022) have been proposed and employed.

Meanwhile, attention mechanism focuses on the important information of an image, which is more expandable and robust (Brauwers and Frasincar, 2021) that improves the performance of CNN models. Different attention modules have been employed. CA is used to learn what information should be focused on, such as in SE-Net (Hu et al., 2018) and ECA-Net (Wang Q. et al., 2020). Split attention is used in SK-Net (Li et al., 2019) and ResNeSt (Zhang et al., 2020) to acquire more comprehensive and reliable CA information. SA is used to learn the position information that should be focused on (Mnih et al., 2014; Li et al., 2018). However, both methods are limited that some important feature information is not fully considered which is useful for CHS recognition. Both CA and SA are used in CBAM (Woo et al., 2018) and DA-Net (Fu et al., 2019). However, fine-grained structures as those in split attention modules are not used to capture more subtle variations in different image classes.

### Chinese Herbal Slices Image Recognition

Despite the rapid progress made by machine learning and deep learning, limited recognition research has been conducted on CHS only in recent years. AlexNet (Huang et al., 2019), VGGNet (Sun and Qian, 2016), GoogLeNet (Liu et al., 2018; Hu et al., 2020), EfficientNet (Hao et al., 2021), and Densitynet (Xing et al., 2020) are directly employed or slightly improved. In these works, an appropriate attention mechanism is not employed by most of the research for better capturing the most important feature information of CHS images.

Some more works can be found in publications in Chinese (Zhang et al., 2021). In addition, there are also some related studies have been carried on the recognition of medicinal plants, branches, and leaves (Sabu et al., 2017; Azadnia and Kheiralipour, 2021; Tassis et al., 2021) with similar image information.

In CHS recognition tasks, inter- and intra-class differences are both great in many occasions. A CNN-based method will have a better recognition performance. Meanwhile, based on data analysis and some of our previous research works (Lu and Wang, 2019; Wang J. et al., 2020), texture features are comparatively critical. Then, in deep network design, modules that pay attention to texture features should be considered and emphasized. To address these key issues in CHS image recognition, a CCSM module based on a deep recognition structure is proposed in this paper. The module employs both CA and SA to acquire both global and local information within the feature maps. A max-pooling layer is also employed to focus on the important texture features of CHS.

### Recognition Structure

**FIGURE 3:**
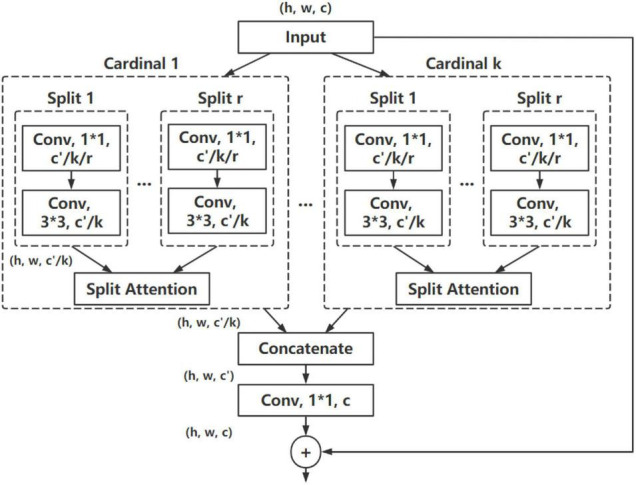
ResNeSt block. *h,w,c* refers to the height, width, and number of channels of the feature map, respectively; *k* is the number of cardinal groups; *r* is the number of splits within a cardinal group.

The recognition structure of the proposed method is based on the ResNeSt (Zhang et al., 2020), which is a ResNet (He et al., 2016) variant with a split-attention block. On the basis of retaining the original ResNet structure, ResNeSt employs group convolution from ResNeXt (Xie et al., 2017) and channel-wise attention mechanism, which enables the information interaction between cross feature map groups through the ResNeSt block, and the feature information can be obtained from different receptive fields. The ResNeSt block is depicted in Figure 3. In this paper, the similar structure of ResNeSt is used, with a combined attention module designed and employed, which is discussed in Section “Combined channel attention and spatial attention module.”

### Combined Channel Attention and Spatial Attention Module

**FIGURE 4:**
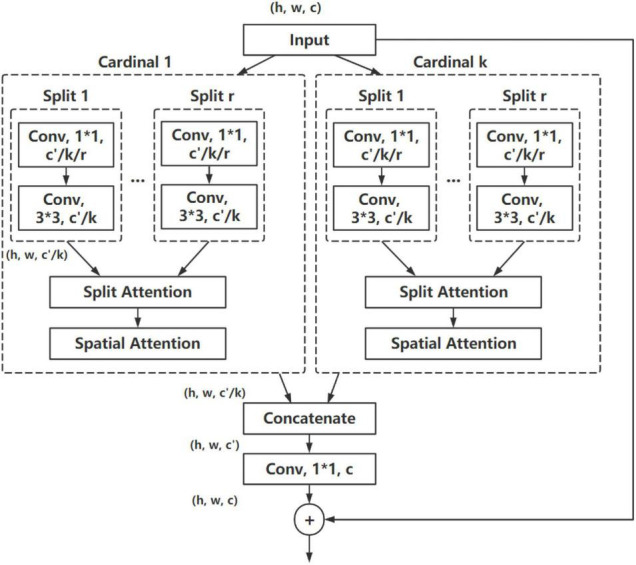
Block with the CCSM module.

In consideration of the characteristic of CHS images, a combined CA and SA module are proposed, as shown in Figure 4. The CCSM is based on ResNeSt structure, while SA is used in addition to the channel-wise attention used in the split attention of original ResNeSt, to focus both the position and essential of the most important information of feature maps. A max pooling is also employed in the module to cover global information of CHS images. The Gaussian Error Linear Unit (GELU) activation function is employed instead of ReLU used in ResNeSt.

#### Channel Attention Module

**FIGURE 5:**
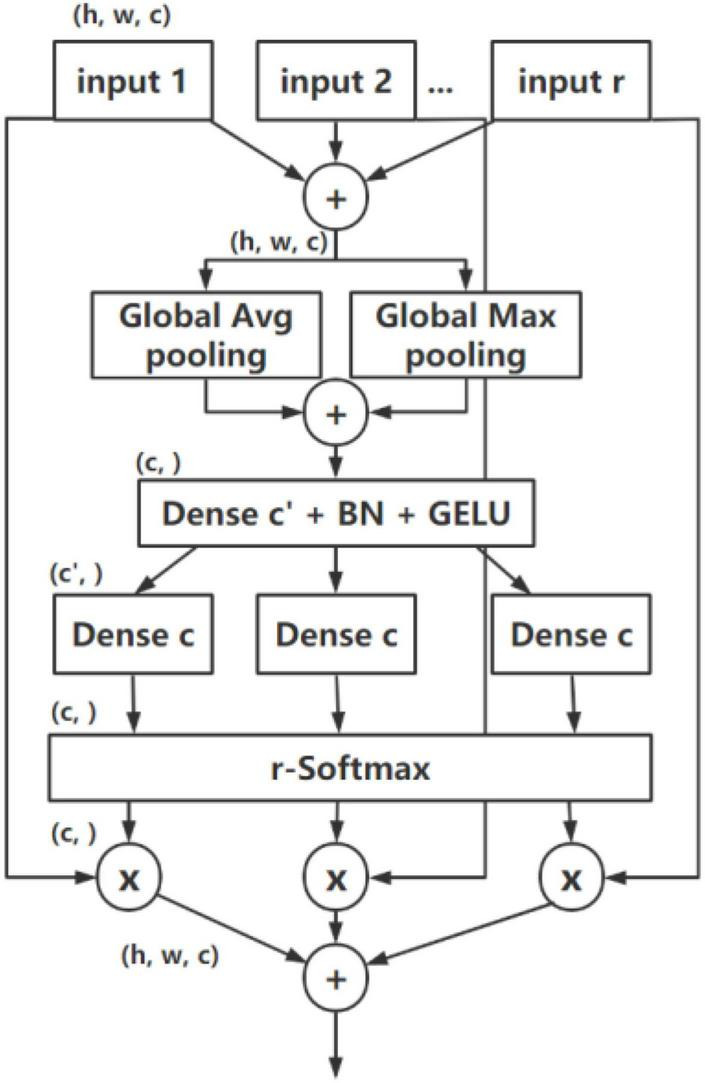
Split attention module.

The proposed CA module is based on the split attention module used in ResNeSt, which is shown in Figure 5. With a similar design, the proposed module employs a split attention block with different pooling design and different activation function.

Due to the variety of texture information in the feature map of CHS, some useless redundant information should be preliminarily removed while keeping important texture features before weight calculation to simplify the feature complexity. Therefore, to get distinctive object features for a finer channel-wise attention (Woo et al., 2018), a max-pooling operation is used and concatenated with a global pooling in the split attention operation in a similar cardinality group of original ResNeSt, which will accurately obtain the global context information, remove the redundant noise information, and better retain and extract the texture features. It can be calculated as:


(1) G(F)=maxPool(F)

where *F* denotes the input feature map.

We use the GELU activation function in the split attention instead of ReLU. As a non-linearity, GELU yields the neuron’s output by multiplying the input by zero or one, but the values of this zero-one mask are stochastically determined while also dependent upon the input, which makes it with better performance than ReLU (Hendrycks and Gimpel, 2016). It is defined as:


(2) $$GELU\left(x\right)=x\cdot \frac{1}{2}\left[1+\text{erf}\left(\frac{x}{\sqrt{2}}\right)\right]$$

(3) $$erf=\frac{x}{\sqrt{2}}\int_{0}^{x}\exp\left(-t^{2}\right)dt$$

where *x* is the neuron input, *erf* is the error function.

#### Spatial Attention Module

**FIGURE 6:**
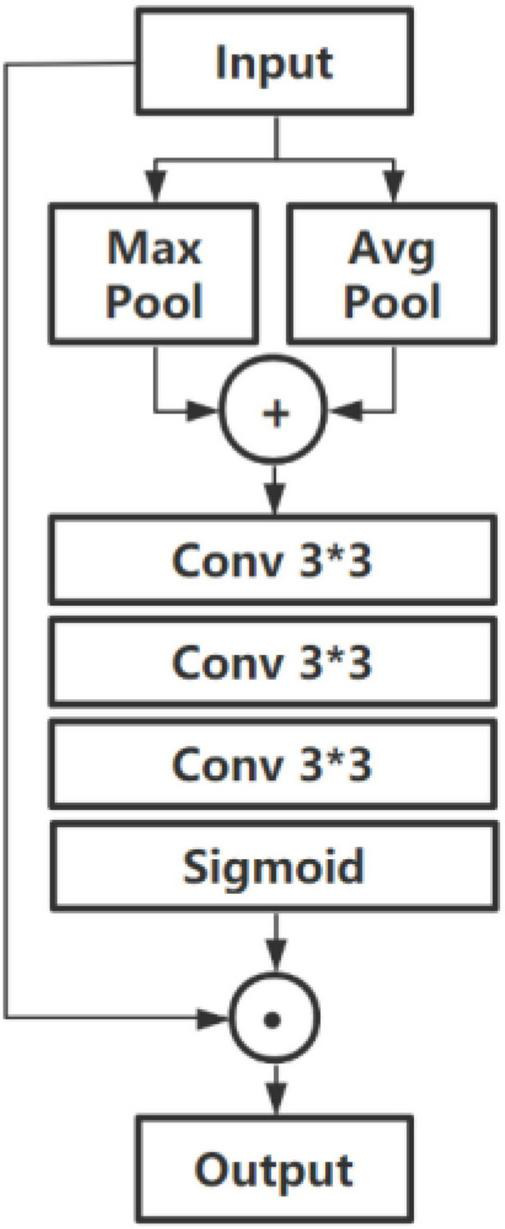
Spatial attention module.

In CHS images, the information importance of different positions of the image is also different. For example, the edge position information of CHS is generally more important than that from other positions. Consequently, the SA is imperative to strengthen such important information. In the split attention module used in ResNeSt, only channel-wise attention is used for acquiring the feature relationship and importance inside the channels. A combined attention module is proposed and employed in this paper, where after a CA, a SA module is also used to generate a two-dimensional SA map. Compared with channel-wise attention, the SA is the supplement and development of CA. It pays more attention to the content information in the spatial position. By distributing the weight in each spatial position, it will be acquired that which spatial position information is most important, and consequently enhance the characteristics of that part of the position, meanwhile inhibiting the extraction of noise features. After the channel-wise attention, a weight-shared SA block is applied to optimize the spatial information. The structure of the SA module is shown in Figure 6.

In the SA module, a pair of max-pooling and average pooling operations are used in the channel axis to aggregate the channel information of the feature map and better retain and extract texture features, which are shown as:


(4) G1(F)=[maxPool(F),avgPool(F)]

where *F* denotes the input feature map, and [⋅] refers to the concatenation operation.

Then after three 3 × 3 convolution operations, the receptive field of the feature map is appropriately expanded. Then a two-dimensional SA map is generated by using a sigmoid function. Thereby, the local information of the feature map is obtained, which is combined with the global information obtained by the CA module. It is computed as:


(5) $$G2\left(F\right)=\sigma \left(f^{3\times 3}\left(f^{3\times 3}\left(f^{3\times 3}\left(F\right)\right)\right)\right)$$

where σ denotes the sigmoid function, and *f*^3×3^ represents the convolution operation with 3 × 3.

Then the weight of the final SA is weighted into the original feature map, which is computed as:


(6) G3(F)=W×F

where *W* denotes the weight acquired by the SA module.

So that the network model extracts diversified features in considering both local and global information, which increases the expression effect of the original image and further improves the classification accuracy.

## Analytical Approaches and Results

### Chinese Herbal Slices Dataset

**FIGURE 7:**
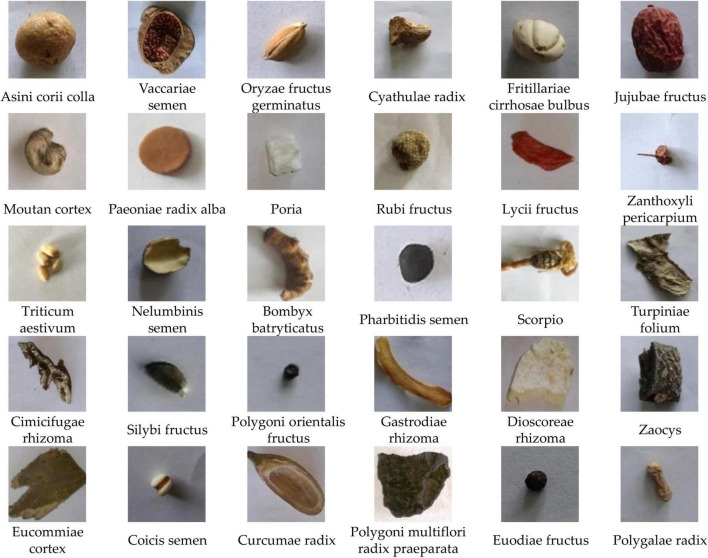
Samples of CHS images in CHSD1.

The images of the dataset are collected from a formal CHS production enterprise under the instruction and inspection of registered Chinese pharmacists. The production and processing follow the Chinese Pharmacopoeia (Chinese Pharmacopoeia Commission, 2020) and the processing specifications of TCM of Zhejiang, China (Zhejiang Medical Products Administration, 2015). The CHSD consists of 2 subsets. One is constructed of single-slice images as CHSD1, as shown in Figure 7. There are 8,886 images with 100 classes. The images were taken by a digital camera in various natural illumination conditions, where pieces with different geometric shapes from every CHS category are shot from different angles by a camera. The single-slice images present the distinct appearance features including shade, texture, and color of every CHS category, which is used for algorithm comparison, selection, and validation in some of our works (Lu and Wang, 2019; Wang J. et al., 2020).

**FIGURE 8:**
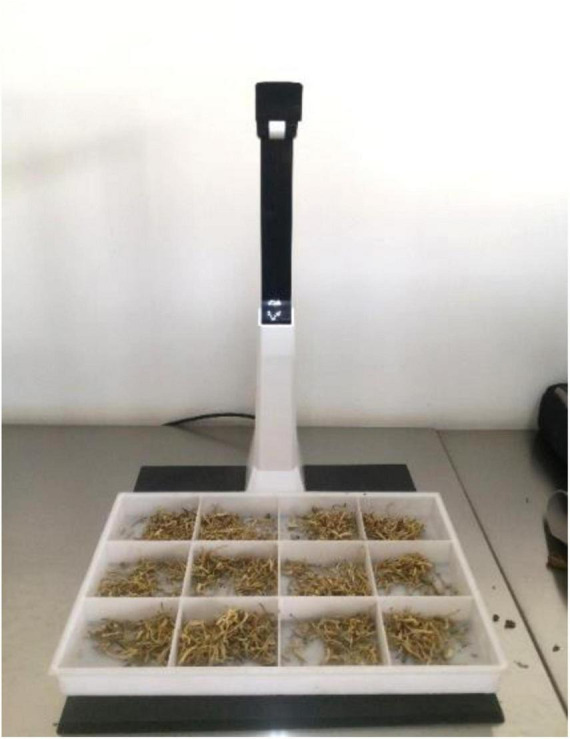
CHS in a sample grid tray.

**FIGURE 9:**
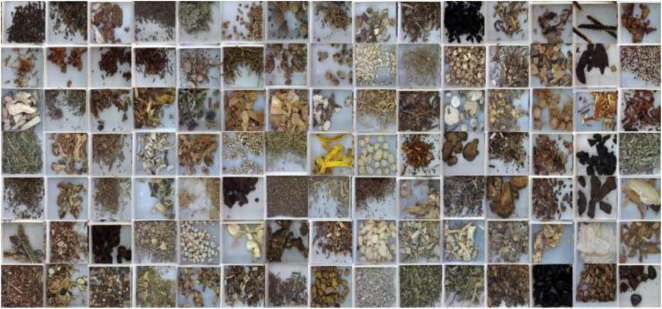
Sample images of CHSD2.

As the research in this paper is aimed to be applicable to real CHS production and dispensing scenario, the images should be consistent with the real recognition process, where in most cases, the samples of every CHS category in each dose of prescription are placed on the sample grid tray, one category in a grid, for identification, as shown in Figure 8. Therefore, another data subset CHSD2 composes images of overlapped CHS in small piles just as those in the sample grid tray, which is used by pharmacists at the dispensing inspection process in TCM decoction and other preparations production. The images of the sample grid tray are taken by a document camera under a uniform illumination condition from the same spot as of CHSD1, where a certain number of pieces from one category are placed in a grid, and then be cropped according to the grid, as shown in Figure 8. There are 14,196 images with 182 classes of commonly used CHS in CHSD2. A full list of CHS in CHSD2 is shown in Appendix A. There are equally 78 images of each category are collected, in consideration of data balancing. Sample images are shown in Figure 9. In order to take the research in real application scenarios, image data are directly used in model training and testing, and no additional data preprocessing works such as resizing or change of illumination are taken.

### Experimental Data

Different experiments are carried out for model comparison, construction, refining, and validation. All the experiments are conducted on the CHSD2 dataset. The original size of the images in CHSD2 shot by the document camera is 2,592 × 1,944 pixels. They are then cropped according to the gird to the size of 648 × 648 pixels each as images of separated piles of CHS, as shown in Figure 9. The dataset is divided into a training set of 80% and a testing set of 20% for experiments. Dataset division is shown in Table 1.

**TABLE 1:** Dataset division.

Dataset	Number of CHS images
Training Set	11,466
Testing Set	2,730
Total	14,196

For the effective comparison with different models such as ResNet or ResNeSt, their general experiment settings are followed. In consideration of the proper numbers of training epochs, without being over-fitting or under-fitting, some pre-experiments have been carried out for testing and validating. According to the results, we set the batch size to 64, the initial learning rate to 0.005, with 150 epochs for all the models training.

For a better assessment of the performance and complexity of the proposed method in comparison with other models, some commonly used metrics such as top-1 accuracy, parameter sizes, macro-precision (*P*), macro-recall (*R*), and macro-*F1* score (*F1*) of each model are used. For the assessment of the performance of data volume on the proposed method, different category sizes of the dataset are used for evaluation. For a fair comparison, all the experiments are conducted with the same NVIDIA Tesla V100-SXM2-32GB GPU and Python version 3.6.9 on Linux operating system. And all the models are produced in the PyTorch framework of version 1.9.0 for training and testing.

### Results

#### Proposed Method With Different Data Settings

Experiments have been taken to test the performance of the proposed method with different data settings. In order to explore the relationship between the number of CHS categories and the recognition accuracy of the proposed method, different numbers of CHS categories are tested in model training and testing. The results are shown in Table 2, where four different numbers of CHS categories of 50, 100, 150, and 182 are compared. Data split is uniformly set to 80% for training and 20% for testing. As shown in the table, when using only 50 CHS categories, the proposed method will reach a high recognition rate of 99.49%, which demonstrate the efficacy of the method. With the number of categories increases, though the image number of the training set increases accordingly, the recognition rate slightly decreases. But it is still steady at a high level, which means that the recognition rate of the proposed model is stable. It shows that the proposed method maintains high recognition rates of above 99.27% and keeps good performance with the increasement of the number of categories.

**TABLE 2:** Recognition rate with different numbers of categories.

Number of categories	Top-1 (%)
50	99.49
100	99.34
150	99.33
182	99.27

All 182 categories of CHS are also employed to test the performance with different data splits of the training and testing sets. The accuracy is evaluated at different epochs, and the results are shown in Table 3. It can be seen that the recognition accuracy increases with the increasement of the percentage of the training set and epoch. When 50% of the data are used for training, the proposed model will get a high recognition rate of 98.77% with 150 epochs, which shows that the model can extract features quickly and reach a high recognition rate. It also indicates that the model’s capability of learning the differences of many CHS categories with only a small number of training data. When the percentage of the training set reaches 80%, the recognition rate tends to be stable. It means that to keep the percentage of training set at 80% will not affect the recognition rate much. Meanwhile, more samples could be divided into the testing set, which will be helpful to improve the generalization performance of the model. As a result, data split of 80% for the training set and 150 epochs is employed for model testing and comparison experiments.

**TABLE 3:** Accuracy of different percentage of training set at different epochs.

Percentage of training set	Epoch				
	30	60	90	120	150
50%	68.02%	92.76%	96.62%	98.70%	98.77%
60%	73.52%	95.01%	97.66%	98.45%	98.97%
70%	83.05%	96.37%	98.00%	98.72%	99.13%
80%	83.17%	96.59%	98.64%	98.92%	**99.27%**
90%	91.45%	97.50%	98.95%	98.95%	99.20%

*Bold values in refer to the results of the proposed module.*

#### Studies on Combined Channel Attention and Spatial Attention Module Components

Experiments are conducted to validate the effectiveness of the components of the proposed CCSM module. We have tested the max pooling, CCSM with ReLU as in original ResNeSt, and CCSM with GELU activation function, and compared them with the original ResNeSt as the baseline. The experimental results are shown in Table 4. It can be observed that with only the max pooling design in split attention module, the result outperforms the baseline, indicating max pooling is helpful in obtaining the global context information to get a better recognition result. When combining CA module with SA module as CCSM, the method achieves much better performance, indicating the combination of both attention modules are effective. When using GELU activation function instead of ReLU, the method achieves further improvement, indicating that GELU is complementary. It is notable that all these are without no significant increasement of the size of parameters. This reveals that the proposed method can obtain better performance with the same size of parameters.

**TABLE 4:** Evaluation results of the module.

Model	Params (*M*)	Top-1 (%)	*P* (%)	*R* (%)	*F1* (%)
ResNeSt101	48.3	98.97	99.05	98.97	98.96
ResNeSt101+ MaxPooling	48.3	99.01	99.09	99.01	99.00
ResNeSt101+ CCSM-ReLU	48.3	99.07	99.15	99.07	99.08
ResNeSt101+ CCSM-GELU	48.3	**99.27**	**99.33**	**99.27**	**99.26**

*Bold values in refer to the results of the proposed module.*

#### Recognition Result of the Proposed Method

After training of the proposed method with combined attention module, the final recognition accuracy of 182 categories of CHS is shown in Table 5. The recognition rates of 166 categories, namely 91.21% categories of CHS are 100%; the 97.80% categories of CHS are over 93.75%; and the recognition rates of some categories are relatively lower.

**TABLE 5:** Recognition rate of different CHS categories.

CHS	Recognition rate (%)
166 CHS categories except as listed below	100
Gnaphalium affine D.don, scorpio, scutellariae barbatae herba, isatidis folium, oryzae fructus germinatus, piperis kadsurae caulis, arcae concha, glycyrrhizae radix et rhizoma, citri grandis exocarpium, hordei fructus germinatus, mori cortex, siegesbeckiae herba	93.75
Eucommiae cortex, angelicae sinensis radix, spatholobi caulis	88.24
Coicis semen	87.50

**FIGURE 10:**
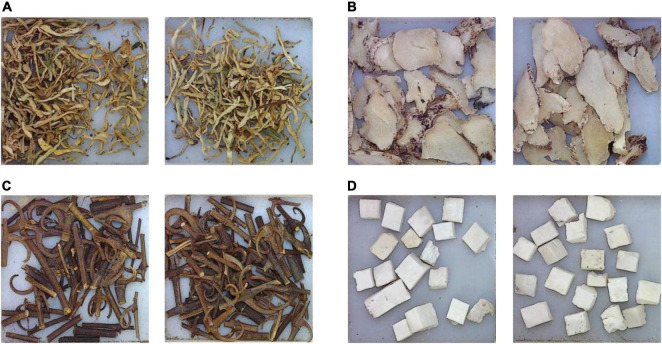
Samples of correctly recognized CHS categories. **(A)** Lonicerae japonicae flos. **(B)** Bletillae rhizoma. **(C)** Uncariae ramulus cum uncis. **(D)** Poria.

**FIGURE 11:**
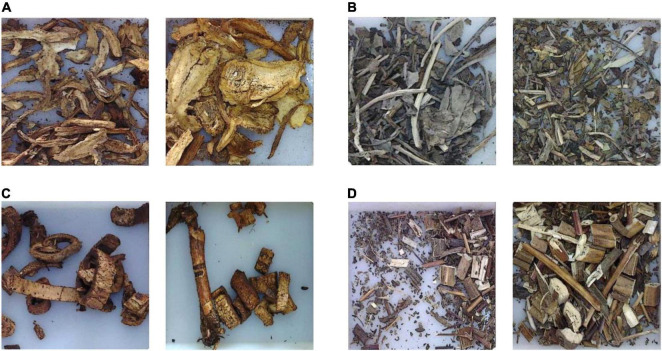
Samples of CHS categories with recognition rate under 100%. **(A)** Angelicae sinensis radix. **(B)** Isatidis folium. **(C)** Mori cortex. **(D)** Siegesbeckiae herba.

**FIGURE 12:**
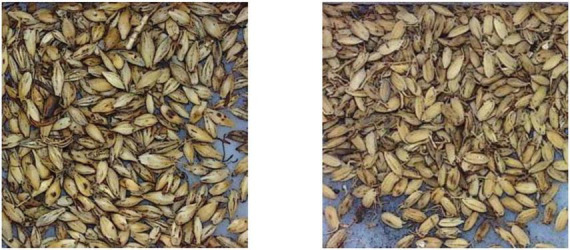
Hordei fructus germinatus **(left)** and oryzae fructus germinatus **(right)**.

**FIGURE 13:**
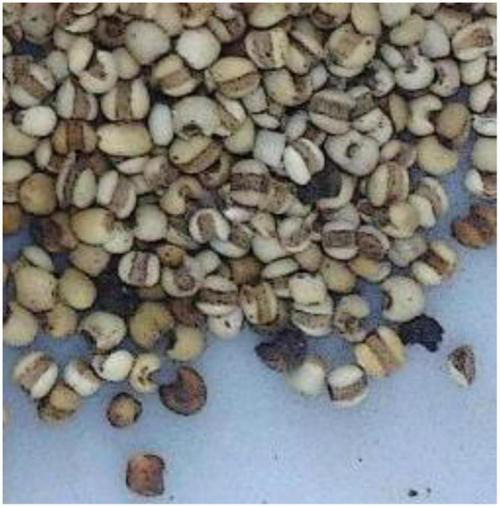
Coicis semen.

It can be found that the correctly recognized categories are always with more uniform appearance features in shape, color, and texture, and are generally easy for the model to learn and recognize, as shown in Figure 10. For those categories with a recognition rate under 100%, after carefully analysis, it is found that the reason lies in two aspects. Firstly, great inner-class differences can be found in some CHS categories, as shown in Figure 11. Differences can be observed in the figure. In some cases, the whole herbs, leaves, small branches, and flowers can all appear in one category. Fragmented pieces can also be seen in some categories. Together with different viewing angles of the pieces, there will be many different features available for the model to learn and recognize. Considering the data volume of the training set, it is difficult for the model to learn abundant feature information, which implies that in the following research, data volume should be expanded to provide sufficient training data. On the other hand, inter-class similarity will also affect the recognition results. The appearance and texture of some categories in the dataset are very similar to each other, such as hordei fructus germinatus and oryzae fructus germinatus, as shown in Figure 12. It is not easy to distinguish from each other by appearance features, which will cause some interference to the recognition results. Among them, the recognition rate of coicis semen is the lowest. Moreover, it is found that there are some blackened slices in the images of coicis semen, as shown in Figure 13. From the analysis of the images and discussion with TCM pharmacists and product quality control professionals, the reason was found may partly lie on that during herbal slices processing, uneven heating of the slices will lead to color variation to different extent. The reason of uneven heating rest with that the temperature control or the frying process control of the herbal slices production process is not well executed or varied during processing. Although these pieces are still valid for use in prescription, their appearance will affect the feature extraction of the model.

At the same time, the different stacking methods of some small pieces of CHS also have an impact on the recognition result. Dense stacking will interfere with the texture extraction of CHS. These suggest that in subsequent data collection, the quantity and quality of the image dataset should be carefully considered for improvement. The training data diversity of different stacking methods with different characters of CHS should be increased by randomly putting different quantities of pieces with different appearances as many times as possible. It should also be mentioned that fragmentation is unavoidable when slicing a herbal into multiple small pieces during manufacturing. However, in practical CHS usage, the fragmented pieces are mixed with the complete pieces together. As in the dataset, the images are taken from stacked piles of the pieces, in data collection, fragmented as well as complete pieces should both be fully collected to cover their different features. Then the dataset will cover the diversified appearance and texture of the CHS, and meet the needs of the application scenarios at the same time. Meanwhile, in real applications from different production environments, CHS images maybe with different sizes or illumination conditions. Appropriate data preprocessing works should also be taken to better increase the generalization ability of the model.

**FIGURE 14:**
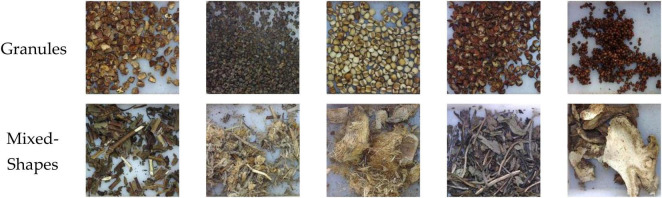
Samples of shape type of granule and mixed-shapes.

Experiments have also been taken on different shape types to verify the efficacy of the proposed method in inter-class recognition. All CHS images in the dataset are grouped into different shape types. As different parts of herbal can be used, there are some categories including different shape types, as shown in Figure 11. As a result, these categories are grouped into a separate type of mixed shapes. Therefore, the images are grouped into 6 shape types, such as pieces, silks, segments, blocks, granules, and mixed shapes, as shown in Figures 1, 14. The percentages of CHS categories for the 6 types range from 7% (silks) to 25% (pieces), which is the typical distribution of all CHS categories. The recognition results are shown in Table 6. The recognition rates of six shape types are all above 96.66%, which shows that the proposed method can classify different shape types efficiently in considering the characteristics of CHS images. The type of granules has the highest recognition rate of 99.78%, and the type of mixed-shapes is with the lowest recognition rate of 99.66%. It is because the types of granules are always directly from the original herbals. As they are small, processing work like slicing is not performed in CHS production. Their appearance features are comparatively constant, which can be easily learned and recognized by the proposed model. The type of mixed-shapes contains multiple shape types. Inner-class differences are greater than those of single shape types. The abundant feature information is not easy for the model to learn in consideration of limited samples in the dataset.

**TABLE 6:** Recognition rate of different shape types.

Shape types	Number of categories included	Percentage of data	Recognition rate (%)
Pieces	45	25%	98.72
Silks	13	7%	99.51
Segments	29	16%	97.88
Blocks	26	14%	98.77
Granules	29	16%	99.78
Mixed-shapes	40	22%	96.66

#### Comparison With Different Convolutional Neural Network Models

**FIGURE 15:**
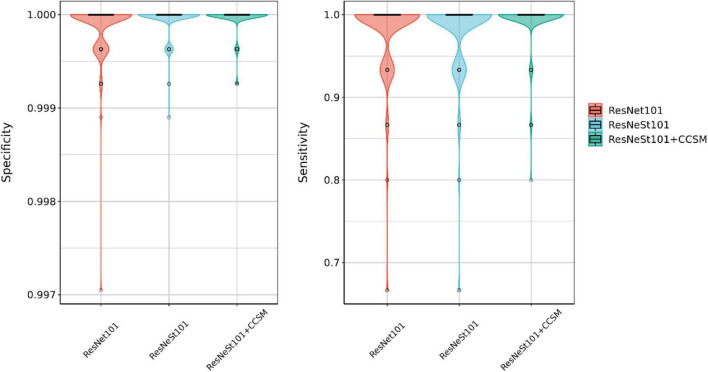
Box plot and violin plot of specificity and sensitivity score.

In the experiments, ResNeSt is used as the base architecture for the proposed CCSM module. In order to assess the performance of the proposed method, different models of ResNet, ResNeXt, and ResNeSt are compared in parameter sizes, top-1 accuracy, precision, recall, and F1-score. Experimental results are shown in Table 7. CCSM module can be applied to different networks, where the results of ResNest50 + CCSM and ResNeSt101 + CCSM are listed in the table. The results with CCSM module are bold. It can be seen from the results that with CCSM module of both CA and SA, the networks’ recognition accuracies are both improved without a significant increasement in parameter sizes. ResNeSt50+CCSM outperforms ResNeSt50 by 0.7% of top-1, 0.6% of precision, 0.7% of recall, and 0.7% of F1-score. ResNeSt101+CCSM outperforms ResNeSt101 by 0.3% of top-1, precision, recall, and F1-score, respectively, and with about the same size of parameters. And the ResNeSt101 + CCSM has the best recognition accuracy of 99.27%, precision of 99.33%, recall of 99.27%, and F1-score of 99.26%. This demonstrates the CA and SA of the CCSM are beneficial to CHS recognition effectively. Figure 15 shows the combination of box plot and violin plot of specificity and sensitivity of ResNet101, ResNeSt101, and ResNeSt101 + CCSM. It can be seen that the result of the proposed ResNeSt101 + CCSM is more concentrated around the area near 1 and there are only a few abnormalities, which indicate that the model has high recognition efficacy in both positive and negative predictions.

**TABLE 7:** Comparison of different residual structure models.

Model	Params (M)	Top-1 (%)	*P* (%)	*R* (%)	F1 (%)
ResNet50	25.5	96.96	97.18	96.96	96.94
ResNeXt50	25.0	98.13	98.28	98.13	98.13
ResNeSt50	27.5	98.53	98.64	98.53	98.52
**ResNeSt50 + CCSM**	27.5	**99.19**	**99.24**	**99.19**	**99.19**
ResNet101	44.5	97.88	98.03	97.84	97.83
ResNeXt101	44.3	98.79	98.87	98.79	98.79
ResNeSt101	48.3	98.97	99.05	98.97	98.96
**ResNeSt101 + CCSM**	48.3	**99.27**	**99.33**	**99.27**	**99.26**

*Bold values in refer to the results of the proposed module.*

## Conclusion

In this paper, combined CA and SA module network (CCSM-Net) for the CHS image recognition are proposed. According to the characteristic of CHS image, in the proposed module, max-pooling with average-pooling is used, and SA is combined with CA to cover the key features for CHS image description. Furthermore, a CHS image dataset is established for recognition research and applications. The images are collected from a local CHS manufacturer in a production environment with 182 CHS classes of 14,196 images. Experiments are conducted based on the dataset to evaluate the efficiency of the proposed method. Results show that the proposed CCSM-Net outperforms various residual structure models with the recognition rate of 99.27%.

## Data Availability Statement

The original contributions presented in this study are included in the article/supplementary material, further inquiries can be directed to the corresponding author/s.

## Author Contributions

JW and WM: conceptualization, methodology, and writing – original draft preparation. WM: software. XX and YL: validation. JW and XX: formal analysis. JW, WM, YW, and JY: investigation. XL: resources and writing – review and editing. XX and YW: data curation. JW, WM, YL, and JY: visualization. JW and XL: supervision and project administration. JW: funding acquisition. All authors read and agreed to the published version of the manuscript.

## Conflict of Interest

The authors declare that the research was conducted in the absence of any commercial or financial relationships that could be construed as a potential conflict of interest.

## Publisher’s Note

All claims expressed in this article are solely those of the authors and do not necessarily represent those of their affiliated organizations, or those of the publisher, the editors and the reviewers. Any product that may be evaluated in this article, or claim that may be made by its manufacturer, is not guaranteed or endorsed by the publisher.

## Appendix

**APPENDIX A:** CHS in CHSD2.

Name of CHS
Caryophylli flos, notoginseng radix et rhizoma, salviae miltiorrhizae radix et rhizoma, mume fructus, ginseng radix et rhizoma, citrusaurantiuml. var.amaraengl., lycopodii herba, citri sarcodactylis fructus, flos citri sarcodactylis, gnaphaliumaffined. don, eupatorii herba, scorpio, semen benincasae, benincasae exocarpium, cassiae semen, pteris multifida poir, siphonostegiae herba, arisaematis rhizoma preparatum, polygonati rhizoma, bupleuri radix, scutellariae barbatae herba, lobeliae chinensis herba, magnoliae officinalis flos, albiziae cortex, albiziae flos, sanguisorba officinalis carbonisatum, kochiae fructus, lycii cortex, sedi herba, arecae pericarpium, sargentodoxae caulis, isatidis folium, rhei radix et rhizoma, rhei radix et rhizoma carbonisatum, gastrodiae rhizoma, gekko, pseudostellariae radix, magnoliae officinalis cortex, cremastrae pseudobulbus pleiones pseudobulbus, crataegi fructus carbonisatum, cyathulae radix, fritillariae cirrhosae bulbus, zingiberis rhizoma, siccus bufo, pogostemonis herba, desmodii styracifolii herba, angelicae sinensis radix, curcumae rhizoma, ficus carical., flos hibisci, oroxyli semen, aucklandiae radix, polygoni perfoliati herba, aurantii fructus immaturus, cinnamomi ramulus, mori folium, platycodonis radix, citri reticulatae semen, citrus tangerine pith, lycopi herba, piperis kadsurae caulis, dendrobii caulis, pumex, cortex erythrinae seu kalopanacis, sepiae endoconcha, lophatheri herba, sojae semen praeparatum, dioscoreae rhizoma, rhapontici radix, aspongopus, codonopsis radix, eupolyphaga steleophaga, sophorae flos, arctii fructus, moutan cortex, vaccariae semen, glycyrrhizae radix et rhizoma, oryzae fructus germinatus, xanthii fructus, raphani semen, vespae nidus, galli gigerii endothelium corneum, hordei fructus germinatus, astragali radix, glycyrrhizae radix et rhizoma praeparata cum melle, hirudo, ostreae concha, arcae concha, magnetitum, lysimachiae herba, spirodelae herba, moutan cortex, moutan cortex carbonisatum, pharbitidis semen, cibotii rhizoma, angelicae pubescentis radix, trichosanthis semen, trichosanthis pericarpium, nardostachyos radix et rhizoma, glycyrrhizae radix et rhizoma, sennae folium, bletillae rhizoma, hedyotis diffusa willd, gleditsiae spina, eucommiae cortex, amomi fructus, centellae herba, spermodermis phaseoli radiati, violae herba, carthami flos, gynostemma pentaphyllum, cinnamomi cortex, arisaema cum bile, sterculiae lychnophorae semen, picrorhizae rhizoma, phragmitis rhizoma, zanthoxyli pericarpium, semen zanthoxyli, citri grandis exocarpium, poria, poriae cutis, abuti lisemen, artemisiae scopariae herba, schizonepetae herba, tsaoko fructus, alpiniae katsumadai semen, hypericum seniavinii maxim., puerariae lobatae radix, taraxaci herba, corni fructus, ligustici rhizoma et radix, polygoni cuspidati rhizoma et radix, scolopendra, mori cortex, cynanchi stauntonii rhizoma et radix, stemonae radix, perillae fructus, asteris radix et rhizoma, myristicae semen, ephedrae herba, cicadae periostracum, rubi fructus, chebulae fructus, eriocauli flos, amomi fructus rotundus, siegesbeckiae herba, dryopteridis crassirhizomatis rhizoma, dryopteridis crassirhizomatis rhizoma carbonisatum, vignae semen, paeoniae radix rubra, plantaginis herba, pheretima, angelicae sinensis radix, trionycis carapax, testudinis carapax et plastrum, paridis rhizoma, tinosporae radix, rosae laevigatae fructus, lonicerae japonicae flos, uncariae ramulus cum uncis, stephaniae tetrandrae radix, saposhnikoviae radix, asini corii colla, citri reticulatae pericarpium, dalbergiae odoriferae lignum, periplocae cortex, alpiniae officinarum rhizoma, euonymus alatus, spatholobi caulis, cervi cornu, cervi cornu degelatinatum, atractylodis rhizoma, coicis semen, ephedrae herba, phellodendri chinensis cortex, scutellariae radix, scutellariae radix carbonisatum, astragali radix, coptidis rhizoma, aconiti lateralis radix praeparata, gentianae radix et rhizoma, and solanum nigrum.

## References

[B1] Alam M. Samad M. D. Vidyaratne L. Glandon A. Iftekharuddin K. M. (2020). Survey on deep neural networks in speech and vision systems. *Neurocomputing* 417 302–321. 10.1016/j.neucom.2020.07.053 33100581 PMC7584105

[B2] Azadnia R. Kheiralipour K. (2021). Recognition of leaves of different medicinal plant species using a robust image processing algorithm and artificial neural networks classifier. *J. Appl. Res. Med. Aromat. Plants* 25:100327. 10.1016/j.jarmap.2021.100327

[B3] Brauwers G. Frasincar F. (2021). *A general survey on attention mechanisms in deep learning.* Piscataway: IEEE. 10.1109/TKDE.2021.3126456

[B4] Chinese Pharmacopoeia Commission (2020). *Pharmacopoeia of The People’s Republic of China 2020.* Beijing: CMSTP Press.

[B5] Fu J. Liu J. Tian H. Li Y. Bao Y. Fang Z. (2019). Dual attention network for scene segmentation, In *2019 IEEE/CVF Conference on Computer Vision and Pattern Recognition (CVPR)*, (Piscataway: IEEE), 3146–3154. 10.1109/TNNLS.2020.3006524

[B6] Gao S. Cheng M. Zhao K. Zhang X. Yang M. Torr P. (2019). Res2Net: a new multi-scale backbone architecture. *IEEE Trans. Pattern Anal. Mach. Intell.* 43 652–662. 10.1109/TPAMI.2019.2938758 31484108

[B7] Gu J. Wang Z. Kuen J. Ma L. Wang G. (2018). Recent advances in convolutional neural networks. *Pattern Recognit*. 77 354–377.

[B8] Hao W. Han M. Yang H. Hao F. Li F. (2021). A novel Chinese herbal medicine classification approach based on EfficientNet. *Syst. Sci. Control. Eng.* 9 304–313. 10.1080/21642583.2021.1901159

[B9] He K. Zhang X. Ren S. Sun J. (2016). Deep residual learning for image recognition, in *2016 IEEE Conference on Computer Vision and Pattern Recognition (CVPR)*, (Piscataway: IEEE), 770–778.

[B10] Hendrycks D. Gimpel K. (2016). *Gaussian Error Linear Units (GELUs).* arXiv:1606.08415 [preprint].

[B11] Hu J. Shen L. Sun G. (2018). Squeeze-and-excitation networks, in *2018 IEEE/CVF Conference on Computer Vision and Pattern Recognition*, (Piscataway: IEEE), 7132–7141.

[B12] Hu J. L. Wang Y. K. Che Z. Y. Li Q. Q. Jiang H. K. Liu L. J. (2020). Image Recognition of Chinese herbal pieces Based on Multi-task Learning Model, in *Conference: 2020 IEEE International Conference on Bioinformatics and Biomedicine (BIBM)*, (Piscataway: IEEE), 1555–1559. 10.1109/bibm49941.2020.9313412

[B13] Hua H. Chung C. (2015). The innovation and modernisation of herbal pieces in China System evolution and policy transitions 1950s–2010s. *Eur. J. Integr. Med*. 7 645–649.

[B14] Huang F. Yu L. Shen T. Jin L. (2019). Chinese herbal medicine leaves classification based on improved AlexNet convolutional neural network, in *Conference: 2019 IEEE 4th Advanced Information Technology, Electronic and Automation Control Conference (IAEAC)*, (Piscataway: IEEE), 1006–1011. 10.1109/IAEAC47372.2019.8997578

[B15] Jing L. Tian Y. (2021). Self-supervised visual feature learning with deep neural networks: a survey. *IEEE Trans. Pattern Anal. Mach. Intell*. 43 4037–4058. 10.1109/TPAMI.2020.2992393 32386141

[B16] Kabbai L. Abdellaoui M. Douik A. (2019). Image classification by combining local and global features. *Vis. Comput.* 35 679–693. 10.1007/s00371-018-1503-0

[B17] Krizhevsky A. (2009). *Learning Multiple Layers of Features from Tiny Images.* Canada: University of Toronto.

[B18] Krizhevsky A. Sutskever I. Hinton G. E. (2012). Imagenet classification with deep convolutional neuralnetworks. *Neural Inf. Pocess. Syst.* 25 1097–1105.

[B19] Li W. Zhu X. Gong S. (2018). Harmonious attention network for person re-identification, in *Conference: 2018 IEEE/CVF Conference on Computer Vision and Pattern Recognition (CVPR)*, (Piscataway: IEEE).

[B20] Li X. Wang W. Hu X. Yang J. (2019). Selective kernel networks, in *Conference: 2019 IEEE/CVF Conference on Computer Vision and Pattern Recognition (CVPR)*, (Piscataway: IEEE). 510–519.

[B21] Lin T. Y. Maire M. Belongie S. Hays J. Zitnick C. L. (2014). Microsoft COCO: common objects in context, *Computer Vision – ECCV 2014. ECCV 2014. Lecture Notes in Computer Science*, eds Fleet D. Pajdla T. Schiele B. Tuytelaars T. (Cham: Springer). 10.1089/big.2021.0262

[B22] Liu S. Chen W. Dong X. (2018). Automatic Classification of Chinese Herbal Based on Deep Learning Method, in *Conference: 2018 14th International Conference on Natural Computation, Fuzzy Systems and Knowledge Discovery (ICNC-FSKD)*, (Piscataway: IEEE), 235–238. 10.1109/FSKD.2018.8687165

[B23] Liu Y. Zhu Q. Cao F. Chen J. Lu G. (2021). High-resolution remote sensing image segmentation framework based on attention mechanism and adaptive weighting. *ISPRS Int. J. Geo-Inf*. 10:241.

[B24] Lu Y. Wang J. (2019). Image recognition on Chinese herbal slices based on HOG-LBP (In Chinese). *Chin. J. Inf. Tradit. Chin. Med.* 26 106–110. 10.1016/j.phrs.2020.104986 32502641

[B25] Mnih V. Heess N. Graves A. (2014). Recurrent models of visual attention. in *Proceedings of the 27th International Conference on Neural Information Processing Systems*, (New York: ACM), 2204–2212. 10.1371/journal.pone.0226880

[B26] Russakovsky O. Deng J. Su H. Krause J. Satheesh S. Ma S. (2015). ImageNet Large Scale Visual Recognition Challenge. *IJCV* 115 211–252.

[B27] Sabu A. Sreekumar K. Nair R. R. (2017). Recognition of ayurvedic medicinal plants from leaves: a computer vision approach, In *2017 Fourth International Conference on Image Information Processing (ICIIP)*, Piscataway: IEEE, 574–578.

[B28] Schmidhuber J. (2015). Deep learning in neural networks: an overview. *Neural Netw.* 61 85–117. 10.1016/j.neunet.2014.09.003 25462637

[B29] Simonyan K. Zisserman A. (2014). Very deep convolutional networks for large-scale image recognition. *arXiv [Preprint].* arXiv:1409.1556

[B30] Sun X. Qian H. (2016). Chinese herbal medicine image recognition and retrieval by convolutional neural network. *PLoS One* 11:e0156327. 10.1371/journal.pone.0156327 27258404 PMC4892594

[B31] Szegedy C. Wei L. Jia Y. Sermanet P. Rabinovich A. (2015). Going deeper with convolutions, in *Conference: 2015 IEEE Conference on Computer Vision and Pattern Recognition (CVPR)*, (Piscataway: IEEE), 1–9.

[B32] Tassis L. M. Tozzi de Souza J. E. Krohling R. A. (2021). A deep learning approach combining instance and semantic segmentation to identify diseases and pests of coffee leaves from in-field images. *Comput. Electron. Agr.* 186:106191. 10.1016/j.compag.2021.106191

[B33] Wang J. Dai K. Li Z. (2020). Deep-Learning based image recognition research on Chinese herbal slices (In Chinese). *Lishizhen Med. Mater. Med. Res*. 31 2930–2933. 32502641

[B34] Wang Q. Wu B. Zhu P. Li P. Hu Q. (2020). ECA-Net: efficient channel attention for deep convolutional neural networks, in *Proc. 2020 IEEE/CVF Conference on Computer Vision and Pattern Recognition (CVPR)*, (Piscataway: IEEE), 11531–11539.

[B35] Woo S. Park J. Lee J. Y. Kweon I. S. (2018). CBAM: convolutional block attention module, in *Computer Vision – ECCV 2018. ECCV 2018. Lecture Notes in Computer Science*, eds Ferrari V. Hebert M. Sminchisescu C. Weiss Y. (Cham: Springer), 3–19. 10.1371/journal.pone.0264551

[B36] Wu P. Li H. Zeng N. Li F. F. M. D. (2022). Yolo: an efficient face mask detection method for COVID-19 prevention and control in public. *Image Vis. Comput.* 117:104341. 10.1016/j.imavis.2021.104341 34848910 PMC8612756

[B37] Xie S. Girshick R. Dollár P. Tu Z. He K. (2017). Aggregated residual transformations for deep neural networks, in *Proceedings of the IEEE Conference on Computer Vision and Pattern Recognition*, (Piscataway: IEEE), 1492–1500.

[B38] Xing C. Huo Y. Huang X. Lu C. Liang Y. Wang A. (2020). Research on image recognition technology of traditional Chinese medicine based on deep transfer learning, in *Conference: 2020 International Conference on Artificial Intelligence and Electromechanical Automation (AIEA)*, (Piscataway: IEEE), 140–146. 10.1109/AIEA51086.2020.00037

[B39] Xu Y. Wen G. Hu Y. Luo M. Dai D. Zhuang Y. (2021). Multiple attentional pyramid networks for Chinese herbal recognition. *Pattern Recognit.* 110:107558. 10.1016/j.patcog.2020.107558

[B40] Zeng N. Wu P. Wang Z. Li H. Liu W. Liu X. (2022). A Small-Sized Object Detection Oriented Multi-Scale Feature Fusion Approach With Application to Defect Detection. *IEEE Trans. Instrum. Meas.* 71 1–14. 10.1109/TIM.2022.3153997

[B41] Zhang H. Wu C. Zhang Z. Zhu Y. Lin H. Zhang Z. (2020). *ResNeSt: Split-attention networks*, [Preprint] Available Online at https://arXiv.org/abs/2004.08955

[B42] Zhang Y. Wan H. Tu S. (2021). Technical review and case study on classification of Chinese herbal slices based on computer vision (in Chinese). *J. Comput. Appl.* 41, 1–12. 10.11772/j.issn.1001-9081.2021081498

[B43] Zhejiang Medical Products Administration (2015). *Zhejiang Traditional Chinese Medicine Processing Specification.* Beijing: CMSTP Press.

